# Spontaneous retroperitoneal bleeding from rupture of a massive renal angiomyolipoma managed with transcatheter arterial embolization

**DOI:** 10.1016/j.radcr.2025.04.127

**Published:** 2025-06-24

**Authors:** Majd Oweidat, Mohammed Alra'e, Ammar W․ M․ Hassouneh, Besan Sarahna, Ibrahim Kattoush

**Affiliations:** a College of Medicine, Hebron University, Hebron, West Bank, Palestine; b Department of Radiology, Princess Alia Hebron Governmental Hospital, Hebron, West Bank, Palestine; c Department of Radiology, Al-Mezan Specialty Hospital, Hebron, West Bank, Palestine

**Keywords:** Acute abdominal pain, Angioembolization, Hematuria, Ruptured renal angiomyolipoma, Transcatheter arterial embolization

## Abstract

Ruptured renal angiomyolipoma (AML) is a rare but potentially life-threatening condition requiring prompt diagnosis and intervention. We report the case of a young female who presented with acute left-sided abdominal pain and gross hematuria. She had a known diagnosis of a large left renal AML. Initial evaluation revealed stable vital signs and mild anemia. Contrast-enhanced computed tomography (CT) confirmed a ruptured AML with an associated hematoma but no active extravasation. The patient was admitted for conservative management and subsequently underwent transcatheter arterial embolization (TAE) 2 weeks later. Under ultrasound guidance, vascular access was obtained via the right common femoral artery, and superselective catheterization of the feeding arteries to the tumor was performed. Embolization was achieved using a Lipiodol–N-butyl cyanoacrylate (NBCA) glue mixture in a 1:5 ratio. Final angiography showed complete devascularization of the lesion. Follow-up contrast-enhanced CT showed hematoma resolution, reduced tumor size, and no evidence of residual bleeding or nontarget embolization. The patient experienced marked clinical improvement and remained stable on follow-up without recurrence of symptoms. This case highlights the importance of considering renal AML in the differential diagnosis of spontaneous retroperitoneal hemorrhage. It also demonstrates the value of imaging in diagnosis and follow-up, as well as the role of TAE as an effective intervention in selected cases while preserving renal function.

## Introduction

Renal angiomyolipoma (AML), first identified by *Grawitz* in 1900, are the most common benign kidney tumors, occurring more frequently in females than males (4:1). These highly vascular masses consist of blood vessels, smooth muscle, and fat with perivascular epithelioid differentiation [1]. While often detected incidentally during imaging, they may present with symptoms like flank pain, hematuria, or retroperitoneal bleeding [2]. AMLs, unlike hamartomas, are true tumors, not disorganized tissue clusters from local trauma or infection [2].

Imaging is vital for diagnosing and managing renal AMLs [3]. A key diagnostic feature of classic AML is the detection of substantial adipose tissue on imaging [4]. Although benign, these tumors may invade perirenal fat, renal sinuses, or nearby structures, with rare cases involving tumor thrombi extending into the vena cava [2,5,6]. Management depends on factors like size, symptoms, and malignancy potential.

Herein, we present the case of an adult female patient who presented with a ruptured renal AML, which was successfully treated with transcatheter arterial embolization (TAE).

## Case presentation

A female patient in her late 20s presented to the emergency department with severe left-sided abdominal pain. The pain was not related to any history of trauma and was accompanied by visible blood in the urine. She reported no prior medical or surgical history and had recently been diagnosed with a large renal AML of the left kidney at another medical facility.

On initial examination, the patient appeared well, albeit in significant pain. Her vital signs were stable, with no evidence of hypertension or tachycardia. A Foley catheter was inserted, revealing gross hematuria without clots but with continuous bloody urine output. Initial management included intravenous administration of 500 mL fluids and intravenous *Acetaminophen*.

Laboratory investigations were performed to assess her condition. Her hemoglobin level was 9.8 g/dL. Other hematological findings included a red blood cell count of 3.77 million/µL, hematocrit of 28.6%, mean cell volume of 75.9 fL, and mean cell hemoglobin of 24.7 pg. All other laboratory parameters were within normal limits.

**Fig. 1:**
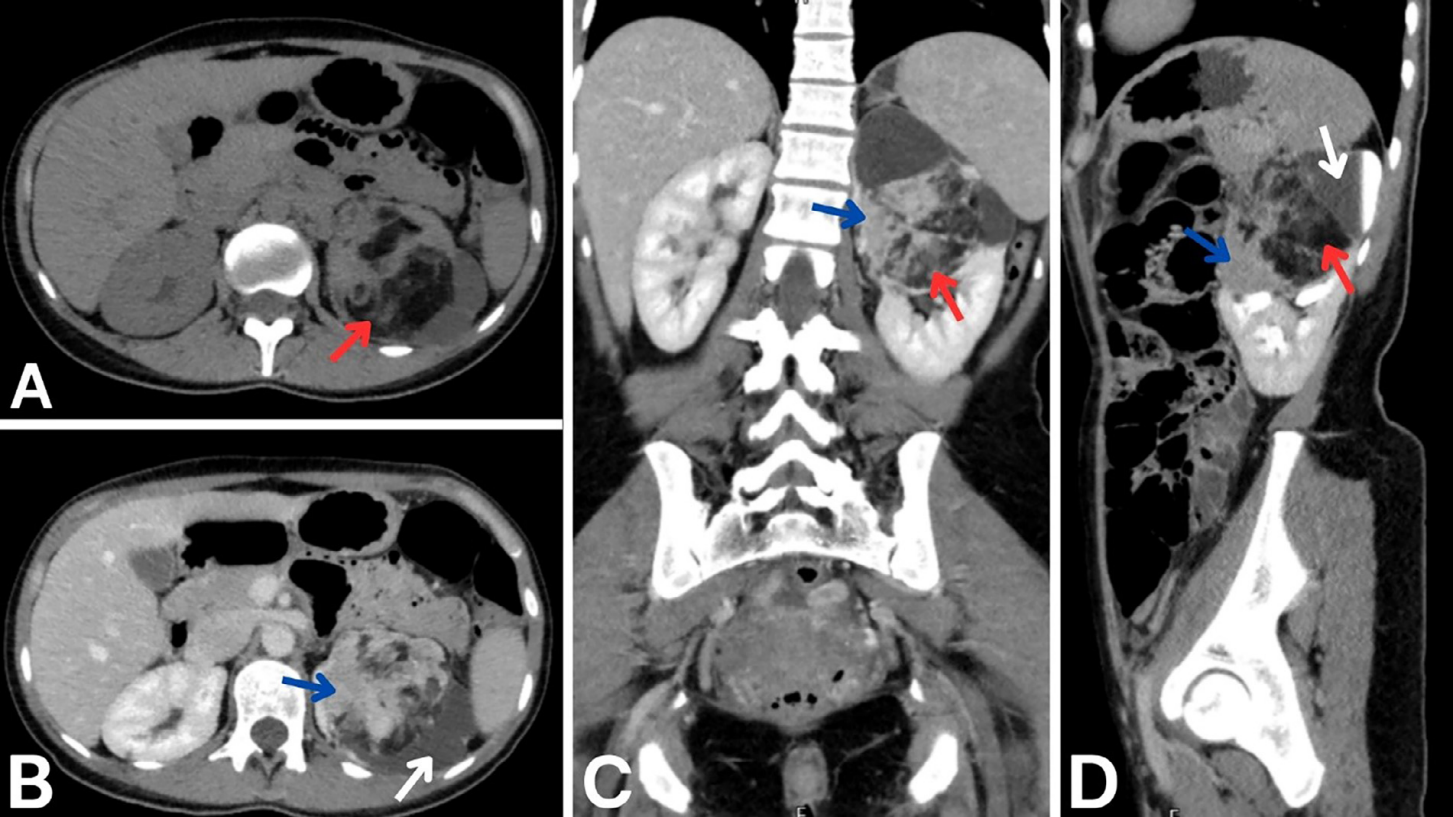
Initial CT imaging with contrast. A: Noncontrast axial plane image showing a large, solid renal mass in the upper and middle poles of the left kidney with mixed densities, including a prominent fat component (red arrow). B: Axial plane in the portal phase showing enhancing soft tissue components (blue arrow) within the mass. Also, it reveals upper calyceal dilation (white arrow) caused by the mass effect from the lesion. C: Coronal plane in the portal phase revealing soft tissue components (blue arrow), and a prominent fat component (red arrow). D: Sagittal plane in the delayed phase highlighting the fat component (red arrow), upper calyceal dilation (white arrow) and soft tissue components (blue arrow).

**Fig. 2:**
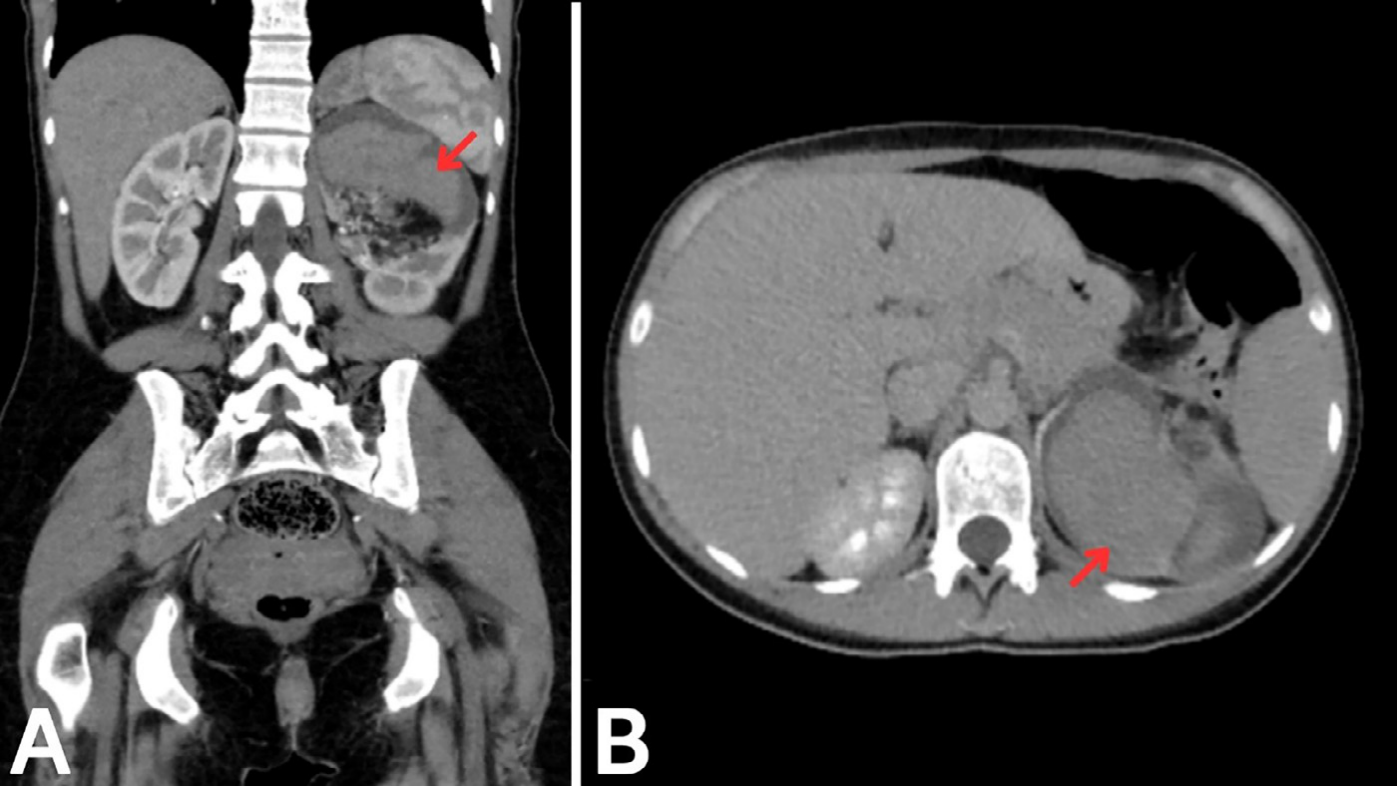
Follow-up CT with contrast showing ruptured AML. A: Coronal plane in the portal phase showing a newly developed hyperdense hematoma (red arrow) within the left renal mass, consistent with rupture. B: Axial plane in the portal phase confirming the presence of the hematoma (red arrow) and its localization within the mass.

A computed tomography (CT) scan of the abdomen and pelvis with contrast shown in Fig. 1, performed at another center approximately 1 month earlier, revealed a large, mixed-density renal mass involving the upper and middle poles of the left kidney. The mass measured approximately 9.0 × 7.5 × 7.3 cm and was noted to have fat components and enhancing soft tissue consistent with AML. There was evidence of upper calyceal dilation caused by the mass, along with thinning of the renal parenchyma. No evidence of metastatic disease or abnormalities in the surrounding structures was observed. Follow-up imaging conducted during the current admission shown in Fig. 2 demonstrated the development of a hyperdense hematoma within the left renal mass, consistent with a rupture of the AML. There was no indication of active bleeding or contrast extravasation.

The patient was admitted and managed conservatively. She was placed on bed rest and closely monitored, with repeated vital sign assessments. Intravenous fluids were administered at a rate of 500 mL over 2-3 hours, and a repeat complete blood count was scheduled for the following morning. Based on the findings and clinical status, the patient was evaluated for possible TAE of the left renal AML.

**Fig. 3:**
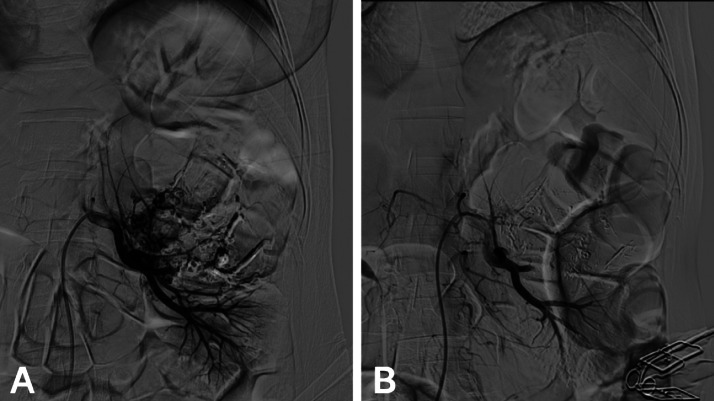
TAE of ruptured left renal AML. A: Digital subtraction angiography reveals 2 feeding arteries supplying the hypervascular AML. B: Postembolization angiographic control following superselective embolization of both branches using a Lipiodol–glue mixture in a 1:5 ratio, showing successful devascularization of the lesion.

**Fig. 4:**
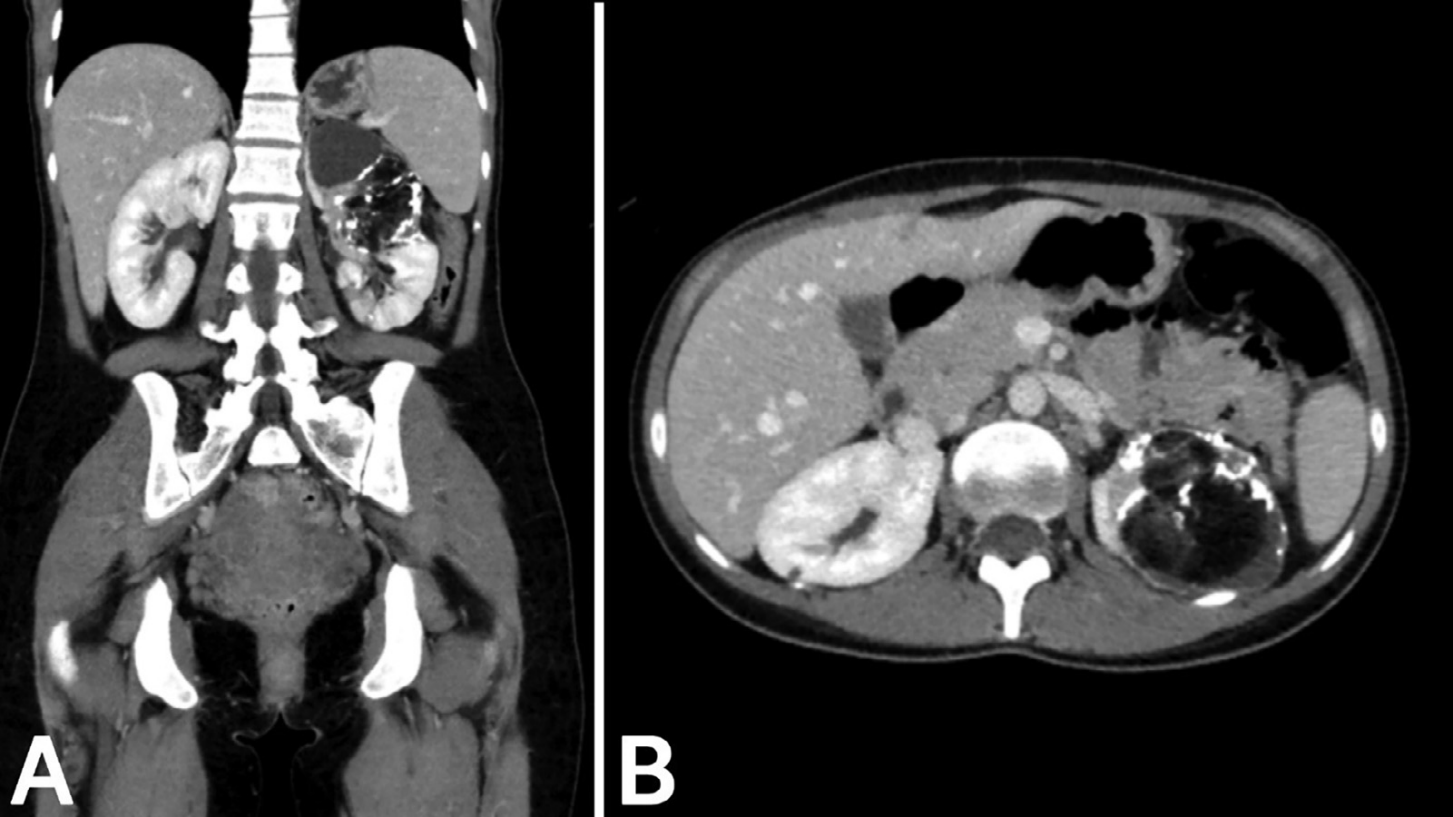
Post-TAE CT with contrast findings. A: Coronal plane in the portal phase showing the resolved hematoma and lipiodol distribution within the AML in the left kidney, confirming targeted embolization. B: Axial plane in the portal phase showing the decreased size of the AML and absence of residual enhancement, indicating successful TAE.

Two weeks later, the patient underwent successful TAE. Under ultrasound guidance, access was obtained via the right common femoral artery using a 6-French vascular sheath. Selective catheterization of the left renal artery was performed. Digital subtraction angiography revealed a large hypervascular mass with abnormal tortuous vessels and a microaneurysm (Fig. 3A). Two feeding arteries supplying the AML were identified and superselectively catheterized. Embolization was achieved using a Lipiodol–N-butyl cyanoacrylate (NBCA) glue mixture at a 1:5 ratio. Final angiography (Fig. 3B) confirmed complete devascularization of the tumor without evidence of nontarget embolization. A follow-up contrast-enhanced CT scan (Fig. 4) showed resolution of the previously noted hematoma and a reduction in the size of the left renal mass to 6.5 × 5 cm. Lipiodol deposition was evident within the lesion, confirming targeted embolization. No residual enhancement or active bleeding was observed. The mass continued to exert pressure on the upper calyceal system, causing persistent but stable dilation.

At her most recent follow-up, the patient reported significant symptom improvement, with no further episodes of hematuria or abdominal pain. She was advised to continue regular follow-up for monitoring of the lesion and renal function, along with adherence to a structured care plan to manage her condition effectively.

## Discussion

This article reports the case of a female patient in her late 20s with a known renal AML, who presented with left-sided abdominal pain. Imaging revealed rupture of the AML, which was subsequently treated with TAE. Postprocedural CT imaging confirmed the success of the treatment.

Renal AML is classified within the family of perivascular epithelioid cell tumors (PEComa). Histologically, AMLs are characterized by a unique triphasic composition which display perivascular epithelioid differentiation [1,6]. Although most AMLs are asymptomatic and discovered incidentally, their potential for spontaneous rupture, which is very rare and can result in life-threatening hemorrhage that demands immediate medical attention due to its significant mortality risk [2,4,7]. The present case exemplifies the rare and critical complication of a ruptured AML, highlighting both the rarity and clinical significance of this condition.

Sporadic AMLs, which constitute approximately 80% of cases, are generally influenced by hormonal factors such as estrogen [8,9]. The pathophysiology of sporadic renal AMLs remains unclear, although all tissue components are thought to originate from pericytes, with endothelial cells developing independently [7]. Tumor size is a critical factor, with masses exceeding 4 cm being at higher risk of spontaneous hemorrhage [2]. In our case, the AML size significantly exceeding the threshold, which likely contributed to the rupture. The resultant hemorrhage, although significant, was not severe enough to induce hemodynamic instability, providing an opportunity for conservative initial management and planned intervention.

Imaging plays a key role in the diagnosis and characterization of AML. In “classic” AMLs, the hallmark feature on imaging is the presence of high amounts of fat, which is readily identified on noncontrast CT scans as hypodense areas. However, atypical presentations such as fat-poor or fat-invisible AMLs can pose diagnostic challenges. Magnetic resonance imaging (MRI) can aid in such cases by detecting subtle fat components [4,10]. In this patient, the initial CT scan confirmed the diagnosis of a classic AML. Follow-up imaging during the current admission revealed a hyperdense hematoma within the mass, indicative of rupture. Notably, ruptured AMLs can complicate imaging interpretation, as hemorrhage may obscure the fat-rich nature of the tumor, potentially mimicking malignancies such as renal cell carcinoma (RCC). In such scenarios, tissue biopsy or surgical exploration may be required [4]. However, in the context of a confirmed AML diagnosis and imaging findings consistent with rupture, the focus of the physicians in this case shifted to managing and preventing the complications may happen.

The management of AML depends on tumor size, symptoms, and the presence of complications. Current guidelines advocate intervention for AMLs larger than 4 cm, those showing rapid growth (>0.25 cm/year), or those associated with complications such as hemorrhage. Pregnant women and women of childbearing age, as in our case, also warrant a lower threshold for intervention due to the potential influence of hormonal fluctuations on tumor growth and vascular integrity [11,12]. TAE has emerged as a preferred treatment modality for symptomatic or complicated AMLs, offering a minimally invasive alternative to nephrectomy [11,12]. TAE achieves hemostasis by occluding the feeding vessels of the tumor, thereby reducing vascularity and mass effect while preserving renal function [2]. In this case, TAE was successfully performed, resulting in resolution of the hematoma and a substantial reduction in tumor size, as confirmed by follow-up imaging.

Only a few case reports in the literature have reported instances of ruptured AMLs. *Tajiri et al.* reported a case of a 70-year-old woman with a previously diagnosed renal AML that remained stable for 2 years before rupturing, leading to hemodynamic instability. Contrast-enhanced CT revealed a large retroperitoneal hematoma (11 × 14 × 20 cm) with active contrast extravasation from a newly developed microaneurysm. Angiography confirmed the bleeding source, which was successfully embolized with an NBCA-lipiodol mixture. Postprocedure imaging showed cessation of hemorrhage and shrinkage of the hematoma [13]. *Abdurabu et al.* reported a case of a 44-year-old male with a ruptured renal AML presenting as a large perinephric hematoma, confirmed on contrast-enhanced CT, which showed an exophytic renal lesion with peripheral vascularity and contrast extravasation. Digital subtraction angiography (DSA) identified abnormal vascular structures, guiding successful angioembolization with coil and gel foam occlusion [14]. *Khor et al.* reported a case of a 30-year-old pregnant woman with a ruptured 76 × 65 mm AML, diagnosed via MRI showing perinephric hematoma and active bleeding. Angiography confirmed abnormal feeding arteries with contrast extravasation, and subsequent angioembolization successfully controlled the hemorrhage while preserving renal function [15]. These cases, alongside our report, highlight the versatility of imaging and TAE in diagnosing and managing AMLs across diverse clinical scenarios, including larger tumors and high-risk patient populations.

This case adds to the limited literature on ruptured renal AMLs in young females, showing the efficacy of TAE as a life- and kidney-preserving intervention. By highlighting a successful conservative-to-interventional approach, our report highlights the importance of early imaging and supports TAE as a nephron-sparing option. In addition to preserving renal function, TAE also mitigates the risk of life-threatening complications such as *Wunderlich syndrome* [15], offering clinicians a practical reference for managing similar high-risk cases.

## Conclusion

This case of a ruptured AML in an adult female patient treated with TAE highlights the importance of considering renal AML as a potential cause of spontaneous hemorrhage in patients presenting with abdominal pain and hematuria. Imaging, close monitoring, and timely TAE can effectively manage rupture while preserving renal function and avoiding unnecessary surgery.

## Ethical approval

Local ethics committees don’t require ethical approval to report such cases.

## Patient consent

The authors declare that they have obtained written informed consent prior to writing the article, including permission for publication of the images and clinical data included herein.
